# Prognostic significance of peritoneal lavage cytology in staging gastric cancer: systematic review and meta-analysis

**DOI:** 10.1007/s10120-017-0749-y

**Published:** 2017-08-04

**Authors:** Sara Jamel, Sheraz R. Markar, George Malietzis, Amish Acharya, Thanos Athanasiou, George B. Hanna

**Affiliations:** Department of Surgery and Cancer, Imperial College London, 10th Floor QEQM Building, St Mary’s Hospital, South Wharf Road, London, W2 1NY UK

**Keywords:** Gastric cancer, stomach neoplasm, Laparoscopy, Peritoneal cytology, Cancer staging, Cancer prognosis

## Abstract

**Background:**

Peritoneal cytology has been used as a part of the cancer staging of gastric cancer patients. The primary aim of this systematic review was to evaluate the value of peritoneal cytology as part of the staging of gastric cancer and survival prediction. The second aim was to establish if positive cytology may be modified by neoadjuvant therapy, to improve prognosis.

**Methods:**

An electronic literature search was performed using Embase, Medline, Web of Science, and Cochrane library databases up to January 2016. The logarithm of the hazard ratio (HR) with 95% confidence intervals (CI) was used as the primary summary statistic. Comparative studies were used, and the outcome measure was survival in three groups: (1) positive versus negative cytology at staging laparoscopy immediately preceding surgery; (2) effect of neoadjuvant therapy on cytology and survival; and (3) positive cytology in the absence of macroscopic peritoneal disease was compared with obvious macroscopic peritoneal disease.

**Results:**

Pooled analysis demonstrated that positive cytology was associated with significantly reduced overall survival (HR, 3.46; 95% CI, 2.77–4.31; *P* < 0.0001). Interestingly, negative cytology following neoadjuvant chemotherapy was associated with significantly improved overall survival (HR, 0.42; 95% CI, 0.31–0.57; *P* < 0.0001). The absence of macroscopic peritoneal disease with positive cytology was associated with significantly improved overall survival (HR, 0.64; 95% CI, 0.56–0.73; *P* < 0.0001).

**Conclusion:**

This study suggests that patients with initial positive cytology may have a good prognosis following neoadjuvant treatment if the cytology results change to negative after treatment.

## Introduction

The main treatment of advanced nonmetastatic gastric cancer is surgical resection with perioperative chemotherapy or chemoradiotherapy [1, 2]. Efforts to prolong survival in metastatic gastric cancer have showed little improvement [1, 2]. Accurate staging of gastric cancer is crucial in selecting the appropriate treatment option, whether curative or palliative. The Japanese Gastric Cancer Association included the results of cytological examination of peritoneal lavage fluid as a key prognostic factor in their classification of gastric carcinoma [1, 3]. However, recently published guidelines suggested that cytology-positive status in the absence of other noncurative factors, that is, macroscopic disease, can be managed with D2 gastrectomy and perioperative chemotherapy [4]. Initial data of those treated with surgery alone showed poor 5-year survival; however, more recent publications have shown that the use of postoperative chemotherapy improves overall survival rates to 26%, [5, 6]. On the other hand, if the information on cytology status were available before surgery, a chemotherapy-first strategy could be taken whereby patients whose cytology status turned negative could be preferentially treated with curative surgery [7, 8].

The incidence of positive peritoneal cytology for patients with gastric cancer varies, in published reports, from 4% to 41% [9]. Peritoneal washings positive for cancer cells have been demonstrated to correlate with the extent of cancer (T1/T2, 0%; T3/T4, 10%; M+, 59%) [10] and have been considered as stage IV disease [11]. The influence of positive cytology on survival has been shown as a powerful independent predictor of survival when compared to other postoperative pathological variables such as the tumor serosal invasion or lymph node involvement [2, 6, 12, 13]. Positive cytology was shown to be the most powerful predictor of outcome, with a risk ratio of 2.7 for patients undergoing curative resection [2]. Furthermore, studies have also shown that the number and arrangement of cytology-positive cells have an effect on survival at the time of gastrectomy [10, 12].

The results of the randomized controlled trial by the apan Clinical Oncology Group (JCOG 0705) and Korea Gastric Cancer Association (KGCA01), comparing gastrectomy plus chemotherapy versus chemotherapy alone in advanced gastric cancer with a single noncurable factor, showed no advantage of resecting the primary gastric cancer in the presence of peritoneal metastasis [11]. Nevertheless, the treatment recommendations for gastric cancer in the event of positive cytology range from palliative chemotherapy to attempts at neoadjuvant therapy followed by surgical resection [4, 14].

The aim of this study was to evaluate the value of peritoneal cytology as part of the staging of gastric cancer and survival prediction. The secondary aim is to establish if positive cytology may be modified by neoadjuvant therapy to improve prognosis.

## Methods

### Literature search strategy

**Fig. 1:**
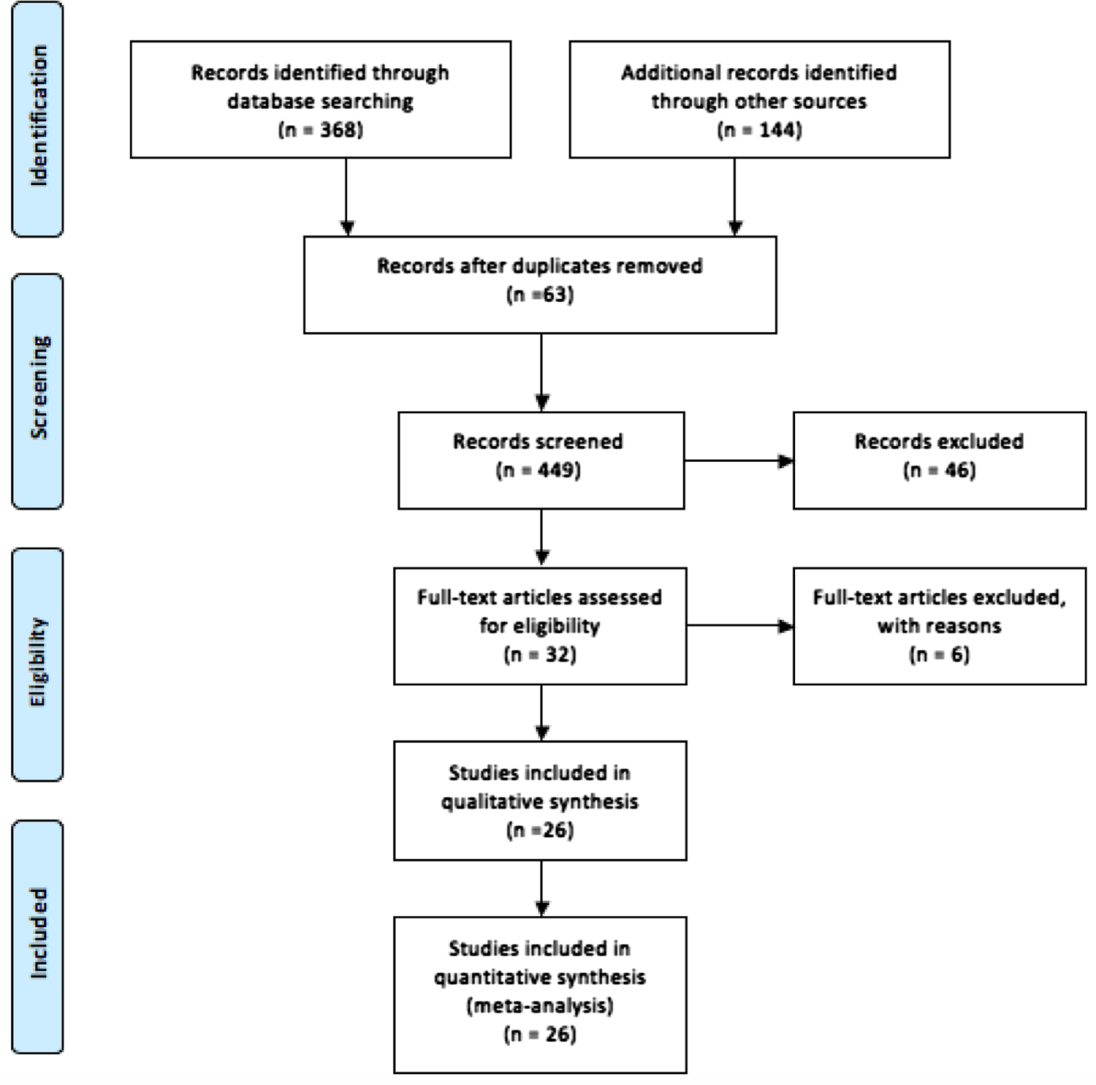
PRISMA flow diagram of literature search in this meta-analysis

An electronic literature search was undertaken using Embase, Medline, Web of Science, and Cochrane Library databases up to January 2016. The search terms ‘gastric cancer,’ ‘laparoscopy,’ ‘peritoneal cytology,’ ‘cancer staging,’ and ‘prognosis,’ and Medical Subject Headings (MeSH) ‘stomach neoplasms,’ ‘neoplasm metastasis,’ ‘laparoscopy,’ and ‘cytology’ were used in combination with the Boolean operators AND or OR (Fig. 1). Two authors (S.J. and S.R.M.) performed the electronic search independently in January 2016. The electronic search was supplemented by a hand-search of published abstracts from meetings of the Society of Academic and Research Surgery, Digestive Disease Week, the Association of Upper Gastro-Intestinal Surgeons of Great Britain and Ireland, and the American Society of Clinical Oncology for 2005–2015. The reference lists of articles obtained were also searched to identify further relevant citations; as was the Current Controlled Trials Register (http://www.controlled-trials.com). Abstracts of the articles identified by the electronic search were scrutinized by two of the authors (S.J. and S.R.M.) to determine their suitability for inclusion in the pooled analysis. Any discordances regarding study inclusion between these two authors were settled in discussion with a third independent author (A.A.). The quality of evidence provided by each study was evaluated using the Oxford levels of evidence-based medicine scoring system (http://www.cebm.net/oxford-centre-evidence-based-medicine-levels-evidence-march-2009).

Publications were included in this review if they meet the following criteria:

- Studies concerning patients with gastric cancer
- Comparative studies of patients with positive and negative peritoneal cytology
- Comparative studies evaluating the effect of neoadjuvant chemotherapy upon patients with positive cytology from gastric cancer
- Comparative studies of patients with positive peritoneal cytology in the absence of macroscopic peritoneal disease and patients with macroscopic peritoneal disease.

Publications were excluded if they met any of the following criteria:

- Studies published in a language other than English
- Case reports or cohort studies including fewer than ten patients
- Noncomparative studies or studies not concerning peritoneal cytology and gastric cancer.

In the situation in which authors from the same institution had published a primary paper and then an updated analysis with a larger patient cohort, the most recent publication was included in the analysis.

### Outcome measures for meta-analysis of comparative studies

The primary outcome measure evaluated was the hazard ratio (HR) for overall survival. Three comparisons were made:

1. Positive versus negative cytology at staging laparoscopy immediately preceding surgery.
2. Initially positive cytology that became negative following neoadjuvant therapy was compared with positive cytology that remained positive despite neoadjuvant therapy.
3. Positive cytology in the absence of macroscopic peritoneal disease was compared with obviously macroscopic peritoneal disease.

### Statistical analysis

The logarithm of the hazard ratio (HR) with 95% confidence intervals (CI) was used as the primary summary statistic. To estimate HR and its variance, this was extracted from the study directly or required additional calculation depending on the method of data being presented: annual mortality rates, survival curves, number of deaths, or percentage of freedom from death [15].

Meta-analysis of data was conducted using a random effects model. Publication bias was explored graphically with funnel plots to detect asymmetry and any outliers. Interstudy heterogeneity was assessed using the chi-square statistic and the *I* ^2^ value to measure the degree of variation not attributable to chance alone: this was graded as low (*I* ^2^ < 25%), moderate (*I* ^2^ = 25–75%), or high (*I* ^2^ > 75%). The significance level was set at *P* < 0.05. Calculations were performed by G.M. and verified by T.A. This study was performed in line with Cochrane recommendations, following the MOOSE guidelines, using appropriate statistical software (STATA/SE12) [16].

## Results

### Patient demographics

The total number of patients included in this meta-analysis was 7970, with a male to female ratio of 1:1.6 (Table 1). The tumor demographics have been described for each group: negative versus positive cytology (Table 2).

**Table 1:** Demographics of patients with cytology-positive (Cyt +ve) and cytology-negative (Cyt −ve) results

Study	Total patients	F:M ratio	Median age	Age range	No. patients	Cyt +ve	Recurrence	No. patients	Cyt −ve	Recurrence
						Comorbidities			Comorbidities	
Badgwell	381	1/1.8	61	24–88	39			223		
Bentrem	371	1/1.2	67	21–88	24			347		
Brito	72	1/1.8	60.4	47.1–73.7	8			64		
Chuwa	142	1/1.8			36	17	6	1C6	17	6
Euanoraster	97	1/1.8			22		22	75		22
Fukagawa	996	1/1.1			225			541		
Jiang	139		57	28–80	38		31	101		31
Kang	75	1.2.1			7		6	68		6
Katsuragi	124	1/2.1			26	32		39	32	
Kodera	70				13			22		
La Torre	64	01/01/01	64.5	29–84	7			57		
Lee	1072				172			900		
Makino	113	1/2.1			35			78		
de Manzon	168	1/1.7			145			23		
Miyashiro	417	1/0.6	61	27–87	25			295		
Nakagawa	100	1/0.6	62	28–83	9			30		
Noda	1474	1/1.9			91			1383		
Ribeiro	201	1/2.2			15			186		
Rosenberg	351	1/1.65			74			272		
Wong	38				31			125		
Total	6465				1042			4935

**Table 2:** Pathological features of gastric cancer in both positive cytology (a) and negative cytology (b)

	Nodal involvement	Primary tumor	(Clinical T disease)	Tumor differentiation		Histological type		Angiolymphatic invasion
		T1–T2	T3–T4	Differentiated	Poorly differentiated	Intestinal	Diffuse	
(a) Positive cytology								
Badgwel				6	30			
Bentrem	150	4	19					
Brito	5	0–2(5), 3–4 (37)				5	3	5
Chuwa	28	0	36					
Euanoraster	22	14		2	20			18
Fukagawa		234	307					
Jiang	29	3	35	14	24	18	20	17
Kang	5	0	7	1	6			
Katsuragi	34	5	41				14	11
Kodera	20	2	20					
La Torre	7	1	6					
Lee	105	6	102	31	141	37	55	
Makino	28		35			5	30	
de Manzoni	13	0	23			9	14	
Miyashiro	25	10	15	7	18			
Nakagawa								
Noda	88	2	89			33	58	41
Riheiro	14	0	15			6	9	
Rosenberg	57	36	38					
Wong	2	0	2	1	1	1	1	4
(b) Negative cytology								
Badgwel				45	165			
Bentrem	14	172						48
Brito	42	5	3			36	26	
Chuwa	44	46	60					24
Euanoraster	61	14	61	25	50			
Fukagawa		6	13					21
Jiang	53	40	61	41	60	46	55	
Kang	46	23	45	25	43			14
Katsuragi	46	47	23			33	37	
Kodera	29	6	31					
La Torre	36	39	18					
Lee	648	368	429	271	629	359	367	
Makino	49		78			35	43	
de Manzoni	77	78	67			83	62	
Miyashiro								
Nakagawa								559
Noda	977	411	972			577	806	
Ribeiro	131	66	120			85	101	
Rosenberg	114	223	49					
Wong								

### Meta-analysis

#### Positive versus negative cytology

**Fig. 2:**
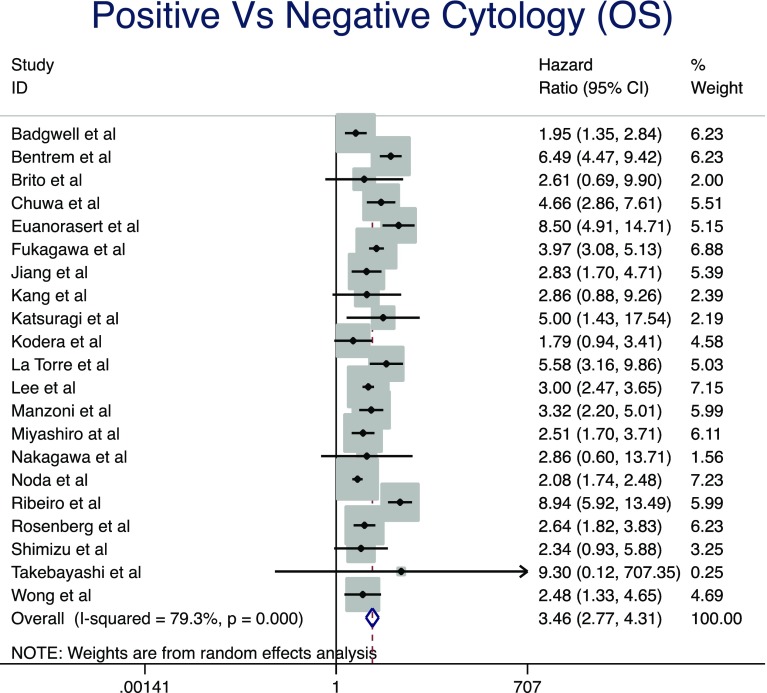
Forrest plot of pooled analysis demonstrated that positive cytology results were associated with significantly reduced overall survival (HR, 3.46; 95% CI, 2.77–4.31; *P* < 0.0001)

Pooled analysis of 21 studies [2, 3, 5, 9, 17–34] included 6499 patients in total, 1052 in the positive and 4948 in the negative cytology group at staging laparoscopy. The median follow-up period ranged from 24 to 140 months. This pooled analysis demonstrated that positive cytology was associated with a significantly reduced overall survival (HR, 3.46; 95% CI, 2.77–4.31; *P* < 0.0001) (Fig. 2). There was evidence of significant statistical heterogeneity for this result (*I* ^2^ = 84.1%).

#### Following neoadjuvant therapy: positive versus negative cytology

**Fig. 3:**
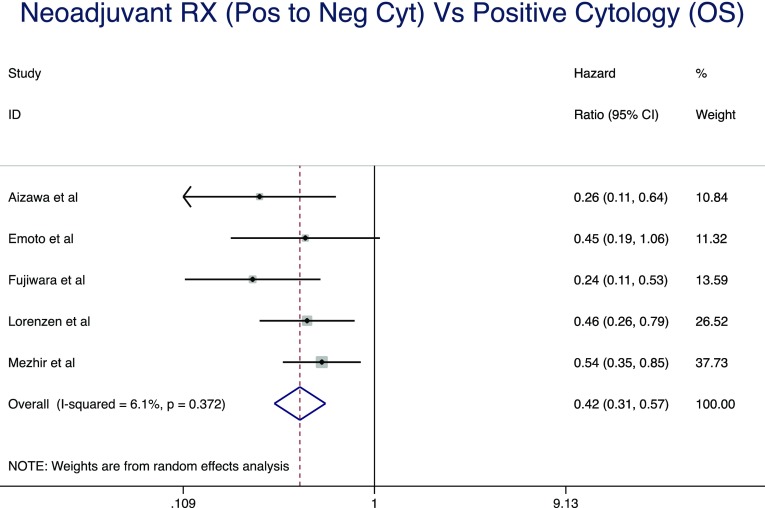
Forrest plot for pooled analysis demonstrated that negative cytology results following neoadjuvant chemotherapy were associated with significantly improved overall survival (HR, 0.42; 95% CI, 0.31–0.57; *P* < 0.0001)

Pooled analysis of five studies [7, 24, 35–37] included 519 patients in total; 73 in the positive cytology and 139 in the negative cytology group. The median follow-up period ranged from 60 to 84 months. This pooled analysis demonstrated that negative cytology following neoadjuvant chemotherapy was associated with significantly improved overall survival (HR, 0.42; 95% CI, 0.31–0.57; *P* < 0.0001) (Fig. 3). There was evidence of significant statistical heterogeneity for this result (*I* ^2^ = 62.5%).

#### Positive cytology versus macroscopic peritoneal disease

**Fig. 4:**
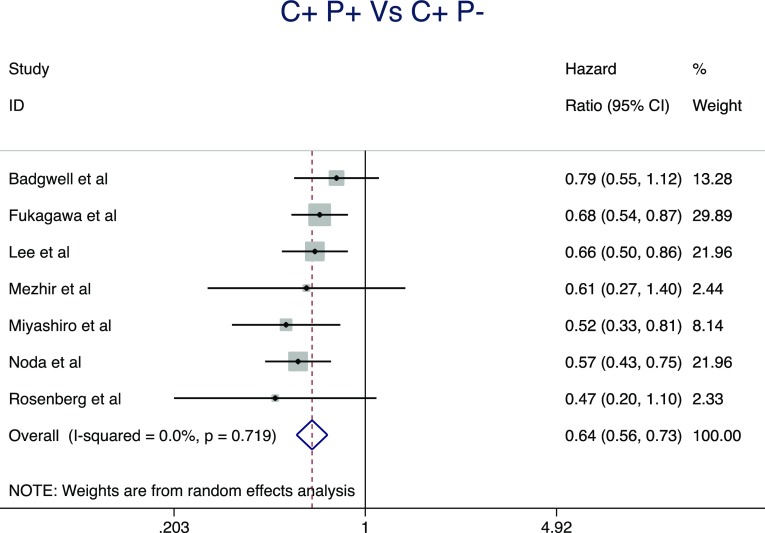
Pooled analysis demonstrated that positive cytology in the absence of macroscopic peritoneal disease was associated with significantly improved overall survival (HR, 0.64; 95% CI, 0.56–0.73; *P* < 0.0001)

Pooled analysis of seven studies [3, 7, 9, 17, 20, 25] included 1035 patients in total, 465 in the positive cytology and 537 in the macroscopic peritoneal disease group. The median follow-up period ranged from 36 to 120 months. This pooled analysis demonstrated that positive cytology in the absence of macroscopic peritoneal disease was associated with a significantly improved overall survival (HR, 0.64; 95% CI, 0.56–0.73; *P* < 0.0001) (Fig. 4). There was no evidence of significant statistical heterogeneity for this result (*I* ^2^ = 0%).

### Bias exploration/sensitivity analyses

Funnel plots were created for combined and subgroup analysis for the various factors to visually assess the publication bias; these demonstrated symmetry. To determine the influence of each study’s individual dataset, we performed sensitivity analyses for the subgroups as described. A single study involved in the pooled meta-analysis was excluded each time, and the corresponding HR was not changed noticeably (data not shown).

## Discussion

This meta-analysis has demonstrated negative peritoneal cytology before treatment improves survival rate when compared with positive cytology. Cytology should be considered a modifiable factor as it was found in this meta-analysis, that the change in cytology status from a positive to negative result following chemotherapy was shown to carry an improvement in overall survival. Furthermore, although positive cytology is considered stage IV disease, the prognosis and overall survival associated with positive cytology versus macroscopic peritoneal metastasis are not equivalent [31]. Fujiwara et al. have shown that the change in cytology status to negative following receiving neo-adjuvant intraperitoneal chemotherapy and systemic chemotherapy improved prognosis [37]. It is worth noting that although Lorenzen et al. have also shown that the change in cytological status to negative following chemotherapy led to improved prognosis, almost 25% with negative cytology became positive following chemotherapy, thus worsening their prognosis and outcome [36]. The prediction of response of patients to neoadjuvant therapy as assessed by cytology remains a challenging area for future research to provide patient- and tumor-targeted therapy.

Regarding peritoneal disease, this meta-analysis has shown that positive cytology alone carries better survival compared with macroscopic peritoneal dissemination. Although survival with no treatment can be similar between these two groups, the use of neo-adjuvant chemotherapy was shown to improve the 3-year overall survival from 0% in gross peritoneal disease to 12% in positive cytology with no overt peritoneal disease [9]. Therefore, the concept that positive cytology is a potentially modifiable factor is further supported. Interestingly, Miyashiro et al. also studied the number of cancer cells detected in cytology studies versus peritoneal metastasis and found that when a higher number of cells was detected, survival was similar to those with peritoneal metastasis [3]. There was insufficient evidence available to define a threshold of positive cancer cells from cytology that changed survival from this review, which remains an important area for future research.

There are a number of limitations to this systematic review and meta-analysis that need to be acknowledged. First, the lack of randomized control trials (RCTs) that met the inclusion criteria is therefore reducing the power of the analyses. Second, only studies in the English-language literature were included, so it is possible that relevant studies in other languages will be identified in the future. Also, patient demographics and co-morbidities were not reported in all the included studies.

**Fig. 5:**
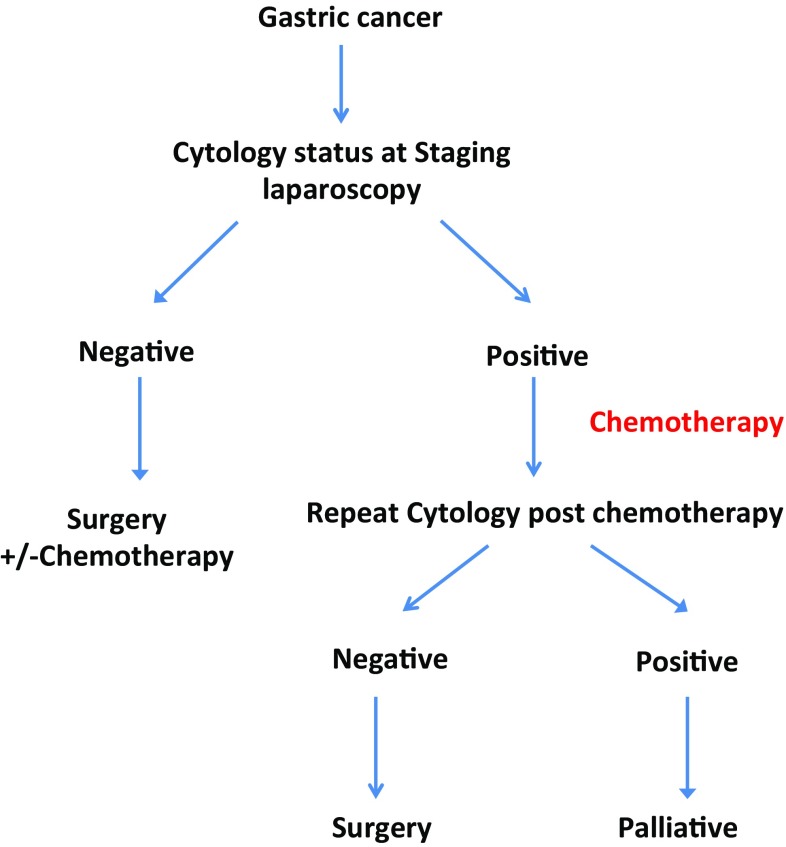
Management algorithm for gastric cancer patients depending on their cytology status and response to chemotherapy

In conclusion, the results of this meta-analysis support the importance of peritoneal cytology results in the assessment of gastric cancer patients. We have shown that negative cytology and the change in cytological status from positive to negative improve survival in gastric cancer. This knowledge justifies the notion to reconsider the presence of positive peritoneal cytology as an absolute indication for palliative intent of treatment without further consideration to changing status following chemotherapy (Fig. 5). The change of initial positive cytology to negative subsequent to treatment should be the subject of a prospective large-scale multicenter study that examines long-term survival benefits.
